# Development of immortalized rhesus macaque kidney cells supporting infection with a panel of viruses

**DOI:** 10.1371/journal.pone.0284048

**Published:** 2023-05-05

**Authors:** Stefanie Reiter, Sabine Gärtner, Katharina Decker, Stefan Pöhlmann, Michael Winkler

**Affiliations:** 1 Infection Biology Unit, German Primate Center, Leibniz Institute for Primate Research, Göttingen, Germany; 2 Faculty of Biology and Psychology, Georg-August-University Göttingen, Göttingen, Germany; The Scripps Research Institute, UNITED STATES

## Abstract

Non-human primate (NHP)-based model systems faithfully reproduce various viral diseases including Ebola, influenza, AIDS and Zika. However, only a small number of NHP cell lines are available and generation of additional cell lines could help to refine these models. We immortalized rhesus macaque kidney cells by lentiviral transduction with a vector encoding telomerase reverse transcriptase (TERT) and report the generation of three TERT-immortalized cell lines derived from rhesus macaque kidney. Expression of the kidney podocyte marker podoplanin on these cells was demonstrated by flow cytometry. Quantitative real-time PCR (qRT-PCR) was employed to demonstrate induction of MX1 expression upon stimulation with interferon (IFN) or viral infection, suggesting a functional IFN system. Further, the cell lines were susceptible to entry driven by the glycoproteins of vesicular stomatitis virus, influenza A virus, Ebola virus, Nipah virus and Lassa virus as assessed by infection with retroviral pseudotypes. Finally, these cells supported growth of Zika virus and the primate simplexviruses Cercopithecine alphaherpesvirus 2 and Papiine alphaherpesvirus 2. In summary, we developed IFN-responsive rhesus macaque kidney cell lines that allowed entry driven by diverse viral glycoproteins and were permissive to infection with Zika virus and primate simplexviruses. These cell lines will be useful for efforts to analyze viral infections of the kidney in macaque models.

## Introduction

The kidney is a target in many systemic viral infections and virus production will result in excretion via urine, a potential route of transmission. Replication of viruses in kidney cells may lead to acute or chronic kidney disease, as reported for example in human immunodeficiency virus (HIV) or Zika virus (ZIKV) infections [1, 2]. In translational research non-human primates (NHP) are important animal models due to their close phylogenetic relationship to humans and physiologic similarities. NHP models of HIV, Ebola virus (EBOV) or ZIKV infection mirror closely the pathogenesis in human patients [3–5]. However, because of ethical considerations, strategies for replacement, reduction and refinement (3R) of NHP models need to be implemented [6]. As a consequence, alternative non-animal methods have been promoted, including but not limited to cell culture models.

Cell lines offer a simple system for analysis of viral infection that combines low cost and ability for upscaling [7]. Usually cell lines are established by explantation of native or tumor tissue and selection of continuously growing cells. To overcome the limited reproductive capacity of native tissue cells, cells can be immortalized using viral or cellular genes like simian virus 40 (SV40) large T [8] or the catalytic subunit of telomerase reverse transcriptase (TERT) [9, 10]. In contrast to the broad availability of human cell lines only a handful of cell lines of NHP origin are available from commercial suppliers (such as ATCC), and only a few additional cell lines have been described in literature [11–13]. Thus, there is a clear need for the development and characterization of new cell lines of NHP origin.

We have established and immortalized, using TERT, novel cell lines derived from rhesus macaque kidney tissue. These cell lines expressed the podocyte marker podoplanin and expressed the IFN-stimulated gene (ISG) MX1 upon stimulation with IFN or viral infection. Finally, these cells were susceptible to infection by viruses from several families, as assessed by pseudotyped viral particles, and supported productive infection with primate herpesviruses and ZIKV. In infection research, these cell lines can be valuable tools for virus isolation and propagation in a species-specific setting, for comparative infection research and in the transition towards NHP models. This last point may also be of general relevance in the study of renal physiology and disease.

## Materials and methods

### Plasmids and oligonucleotides

Plasmids MLV-gag/pol, MLV-luc, Sgpdelta2 and pHIT/G have been described [14–16]. Plasmid HIV-gag-pol was a kind gift from Thomas von Hahn. Plasmids for expression of glycoproteins from Vesicular stomatitis virus (VSV-G), Zaire ebolavirus (EBOV-GP), Nipah virus (NIV-F and NIV-G), influenza A virus strain WSN (IAV HA and NA) and Lassa mammarenavirus (LASV-GPC) have been described previously [16–20]. Oligonucleotides (Table 1) were purchased from Sigma-Aldrich (Steinheim, Germany).

**Table 1:** Oligonucleotides used for cloning.

Name	Sequence 5’-3’
EMCV_IRES3	GCTGAAGGATGCCCAGAAGG
HCMVep-seq	GCAAATGGGCGGTAGGCGTG
hsCA-for	CAGGTCAGCCAAAATTACCCAGTACAACAAATAGGTG
hsCA-rev	CACCTATTTGTTGTACTGGGTAATTTTGGCTGACCTG
Hygro3Mlu	GCCACGCGTTCCGGATTAAACTCGACCTA
polEcoRV-rev	AGGCTCTAAGATTTTTGTCATGC
shCA-for	CAAGCTAGTGCTGAAGGGGTTGGGACCAGGAGCGACAC
shCA-rev	CAAGCTAGTGCTGAAGGGGTTGGGACCAGGAGCGACAC

Retroviral vector pQCXIHy-hTERT was generated by subcloning of hTERT as PmlI/SalI fragment from pBabehygro-TERT (kind gift from Parmjit Jat) [10] into pQCXIHy-mcs [21] cut with HpaI and XhoI. This vector has a shortened C-stretch at the 5’ end of the internal ribosomal entry site (IRES) and the modified multiple cloning site of pQCXIP-mcs [22] which was ultimately derived from pQCXIP (Clontech, Palo Alto, CA, USA). Finally, pQCXIP was modified by mutating an AarI site within the IRES sequence and including AarI, NheI and XbaI sites in the multiple cloning site, to give pQCXIdAP-mcs2.

For lentiviral transduction, a pReceiver-Lv205-based expression plasmid (Genecopoeia, Rockville, MD, USA) was modified stepwise to harbor a pQCXIP-based expression cassette. First, a NdeI/Pfl23II fragment from pQCXIP-PB1 was inserted into EX-A2639-Lv205 to give pLenti-PB1. Second, a NdeI/Acc65I from pQCXIdAP-mcs2 was inserted into pLenti-PB1 to replace the PB1 gene with a multiple cloning site. The resulting vector, termed pLenti-IP-mcs, contained human cytomegalovirus (HCMV) enhancer/promoter, multiple cloning site, internal ribosomal entry sequence and puromycin resistance gene. The multiple cloning site had restrictions sites for MunI-AgeI-PmlI-XbaI-XhoI-BamHI-NotI. To obtain pLenti-IHy, harboring a hygromycin resistance gene, the puromycin resistance gene was replaced by an Acc65I/MluI digested PCR fragment amplified from pQCXIHy-mcs with primers EMCV_IRES3 and Hygro3Mlu. The human TERT gene was then subcloned from pQCXIHy-hTERT into pLenti-IHy-mcs as an AgeI/BspEI fragment to give pLenti-IHy-TERT.

To generate an HIV-based system with improved transduction of rhesus monkey cells, the sequence encoding amino acids 1–204 of the HIV capsid (hCA) was replaced with the sequence encoding amino acids 1–202 of SIV capsid (sCA), giving rise to plasmid HIV gag-pol(SCA) [23, 24]. For this, the flanking HIV parts were amplified by PCR using primers HCMVep-seq/hsCA-rev and shCA-for/polEcoRV-rev and HIV-gag-pol as template, while the sCA was amplified from Sgpdelta2 using primer hsCA-for/shCA-rev2. All the fragments were combined by splice overlap PCR and cloned as EcoRI/EcoRV into HIV-gag-pol. All PCR-amplified sequences were verified by automated sequence analysis.

### Cell culture

Cell lines 293T (DSMZ ACC 635, Braunschweig, Germany), Vero76 (ATCC CRL-1587; kind gift by A. Maisner) and A549 (ATCC CCL-185) were cultivated in DMEM supplemented with 10% fetal calf serum (FCS) and penicillin/streptomycin. Identity of human cell lines was verified by short tandem repeat (STR) analysis [25]. Hybridoma cell line D1-4G2-4-15 was purchased from LGC/ATCC (Teddington, UK) and cultivated in RPMI1640 supplemented with 10% FCS and Pen/Strep.

### Establishment of primary cultures

Kidney cell lines were established from kidney tissue obtained from adult male (6 years, animal 2345) and female (9 years, animal 8639) animals. Kidney tissue from individual samples was chopped into small (1 mm) pieces and seeded into individual 6-well plates in DMEM supplemented with 10% FCS and penicillin, streptomycin, gentamycin, nystatin and amphotericin B. After outgrowth in the course of about two weeks, cells were separately transferred to 25cm^2^ and later 75cm^2^ flasks. Cell line MamuK8639 was derived from the female animal, while cell lines MamuK2345C and MamuK2345MW were derived from the male animal and the cell lines were kept separate at all times.

### Virus

The primate herpesviruses Cercopithecine alphaherpesvirus 2 (CeHV2, SA8) strain B264 and Papiine alphaherpersvirus 2 (PaHV2, HVP2) strain X313 were a kind gift by David Brown and Matthew Jones, Public Health England. ZIKV strain SPH2015 was cloned from RNA and recovered from transfected Vero76 cells [26]. Viruses were propagated on Vero76 cells at low multiplicity of infection (MOI) and harvested when complete cytopathic effect had developed. The recombinant Vesicular stomatitis virus VSV ncp* harboring four amino acid changes associated with reduced cytotoxicity (ncp) and eGFP as reporter (the asterisk stands for eGFP) has been previously described [27].

### Retroviral transduction for the generation of stable cell lines

For production of Lentivirus-based transducing virus, 293T cells were seeded in T25 flasks (at about 10^6^ cells/flask) and transfected on the next day with 6 μg vector pLenti-IHy-TERT along with 3 μg HIV gag-pol(SCA) and 3 μg VSV-G expression plasmid (pHIT/G), employing the Calcium-phosphate co-precipitation method as described before for MLV-based transduction [28, 29]. After incubation with the co-precipitate for 8 h, medium was replaced. Three days after transfection, cell culture supernatants were harvested, filtered through 0.45 μm filters and stored at -80°C.

For transduction and selection we followed a recently established protocol [30]. Thus, cells were seeded in a 96 well plate at 5.000 cells per well. On the next day, 50 μl of transducing virus was added followed by spinoculation at 4,000 × g for 30 min. After two days, cells were transferred into a 24 well plate and selection medium containing 250 μg/mL hygromycin.

### Analysis of viral entry using pseudotyped retroviral particles

Retroviral pseudoparticles were produced in 293T cells essentially as described for transducing retroviruses [28, 29]. Using the Calcium-phosphate co-precipitation transfection method, cells were co-transfected with 6 μg vector MLV-luc, 3 μg MLV-gag-pol and 3 μg expression plasmid for the desired viral glycoprotein. Individual samples expressing no glycoprotein (transfected with empty vector pCAGGS) or VSV-G, EBOV-GP, LASV-GPC, Nipah virus glycoprotein (NIV-G) or influenza A virus hemagglutinin and neuraminidase (IAV-HA/NA) were prepared. After incubation with the co-precipitate for 8 h, cells were replaced in fresh medium and supernatants harvested after additional incubation for three days. Harvested supernatants were filtered through 0.45 μm filters and stored at -80°C.

Target cells were seeded in 96 well plates at 10.000 cells per well. After overnight incubation, medium was replaced with 50 μl fresh medium. For infection, 50 μl pseudovirus containing supernatant was added in triplicate samples and incubated for 4 h, after which an additional 100 μl medium were added. After 72 h, medium was removed and cells were lysed in 50 μl Luciferase Cell Culture Lysis Reagent (Promega, Madison, WI, USA). Luciferase activity was measured in a Plate Chameleon V (Hidex, Turku, Finland) microplate reader employing Beetle-Juice (PJK Biotech, Kleinblittersdorf, Germany) as substrate.

### Induction of MX1 and IFNB1 expression

For analysis of the IFN system, cells were seeded in 12-well plates and either treated with IFN or infected with VSV ncp*, which induced high amounts of IFN [27]. For IFN treatment, cells were incubated for 24 h with medium containing 100 U/mL universal type I IFN-α (pan-Interferon; 11200–2, PBL Assay Science, Piscataway), a chimeric interferon constructed from human IFN alpha A and alpha D [31]. For infection, cells were inoculated with medium containing VSV ncp* at MOI 0.1. After 24 h cells were harvested for RNA isolation and quantitative PCR as described below.

### Virus replication kinetics and titration

For single step growth kinetics cells were seeded in 24 well plates at 50.000 cells/well and infected on the next day with MOI 1 of the respective viruses. For infection, medium was removed and 500 μl inoculum added for an 1 h incubation at 37°C. After removal of inoculum, cells were washed with phosphate-buffered saline (PBS) and finally incubated in 500 μl culture medium. Cell culture supernatant was harvested at fixed time points (1, 24, 48, 72 and 96 h post infection), centrifuged at 4000 rpm for 5 min to pellet floating cells and the cleared supernatant was frozen at -80°C.

Titration of herpesviruses by plaque assay was essentially performed as described previously [32]. Titers of ZIKV were determined in a modified focus forming assay originally developed for IAV [28, 33]. Briefly, Vero76 cells were seeded in 96 well plates at 20.000 cells per well. On the next day, medium was removed and 100μl of tenfold dilutions of virus containing supernatant was added for 1 h at 37°C. After removal of inoculum, 100 μl DMEM medium containing 1% methyl cellulose was added and cells incubated for 72 h at 37°C. Then, the methyl cellulose containing medium was removed and cells washed 1–2 times with PBS. Cells were fixated with cold methanol (10 min -20°C), dried and, after rehydration with PBS, quenched (PBS, 0.5% triton, 20mM glycin) and blocked (PBS, 0.5% triton, 1% BSA). Subsequently, cells were incubated with 50 μl/well hybridoma 4G2 supernatant followed by 50μl secondary anti-mouse horseradish peroxidase (HRP)-conjugated antibody (1:1000, Dianova, Hamburg, Germany). Antibody incubation steps were followed by washing steps. Finally, wells were reacted with TrueBlue peroxidase substrate (Seracare, Milford, MA, USA) until blue foci developed, which were counted, Viral titers were calculated and expressed as focus forming units per milliliter (ffu/mL).

### Quantitative real-time PCR

RNA for quantitative real-time PCR (qRT-PCR) was isolated using the RNeasy Mini Kit (Qiagen, Hilden, Germany) according to the protocol of the manufacturer and eluted in a final volume of 25 μl RNase-free water. To remove contaminating DNA, 1 μg RNA was treated with 0.5 U RNase-free DNase I for 10 min and the reaction stopped by addition of EDTA (final concentration 5 mM) and heating to 75°C for 10 min. For cDNA synthesis, 8 μl of DNase-digested RNA were reverse transcribed using random hexamers and the SuperScript™ III First-Strand Synthesis System (Thermo, Waltham, MA, USA) according to the protocol of the manufacturer. Then 1 μl of the cDNA preparation were used for quantitative PCR using a QuantiTect SYBR Green PCR kit (Qiagen, Hilden, Germany) and the Rotorgene Q platform (Qiagen, Hilden, Germany). Primers against MX1 (forward 5’- TTCAGCACCTGATGGCCTATC-3’, reverse 5’- TGGATGATCAAAGGGATGT-GG-3’), IFNB1 (forward 5’- CAGCAATTTTCAGTGTCAGAAGC-3’, reverse 5’- TCATCCTGTCCTTGAGGCAGT-3’) and the housekeeping gene 18S rRNA (forward 5’-GATCCATTGGAGGGCAAGTCT-3’, reverse 5’-CCAAGATCCAACTACGAGCTT-3’) were previously described [34–36]. Induction of MX1 and IFNB1 expression was analyzed by the 2^-ΔΔCT^ method [37] using 18S rRNA as reference gene.

### Flow cytometry

For flow cytometry (FC), 100.000 cells/well were seeded in 12-well plates, grown overnight and detached with PBS/5 mM EDTA. After centrifugation (1200 rpm, 5 min) cells were resuspended and washed once in FC buffer (PBS, 5% FCS, 2 mM EDTA). After removing the supernatant cells were stained with a rat anti-podoplanin antibody (1:100; Origene AM01133PU-N, Rockville, MD, USA) or rat IgG2a isotype control antibody (Antikörper-online ABIN5675832, Aachen, Germany) in a volume of 50 μl for 30 min on ice. After three washing steps with FC buffer, cells were reacted with secondary Alexafluor488-conjugated donkey anti-rat antibody (1:100; Invitrogen, Carlsbad, CA, USA). After three washes in FC buffer cells were fixated in 2% paraformaldehyde (PFA). Cells were analyzed in a LSR II flow cytometer (Becton, Dickinson, East Rutherford, NJ, USA) using FACS Diva software or an ID7000 Spectral Cell Analyzer (Sony, San Jose, CA, USA). Diagrams for figures were prepared using FlowJo version 10.8.1 (BD BioSciences, Ann Arbor, MI, USA).

### Cell proliferation assay

To analyze cell proliferation, we used the carboxyfluorescein succinimidyl ester (CFSE) Cell Division Tracker Kit (BioLegend, San Diego, CA, USA). In this assay fluorescent CFSE is reacted with cells and thereby covalently attached to cellular molecules. During subsequent cell divisions labelled molecules are transferred to daughter cells, resulting in decreased fluorescence intensity per cell as determined by FC. Cells (250.000) were centrifuged (5 min, 300 ×g) and the cell pellet resuspended in 1 mL CFSE working solution (PBS containing 2.5 μM CFSE). After incubation for 20 min at 37°C in the dark the cell suspension was diluted with 6 mL cell culture medium to stop the reaction. Cells were again centrifuged, resuspended in 2.5 mL cell culture medium and seeded in 24 well plates (50.000 cells in 0.5 mL per well). Cells were harvested immediately after staining or after incubation for 24–96 h. In parallel, control cells were treated with PBS (without CFSE) and harvested immediately or after 72 h incubation at 37°C. For harvesting, cells were detached, centrifuged and resuspended in FC buffer. Cells were then fixated by addition of an equal volume of 4% PFA and stored at 4°C until measurement in an ID7000 Spectral Cell Analyzer (Sony, San Jose, CA, USA). Results were analyzed using FlowJo version 10.8.1 (BD BioSciences, Ann Arbor, MI, USA).

### Microscopy

Phase contrast images were taken at 20x magnification (20x/0.40 HC PL FLUOTAR L objective) on a Leica DMi8 (Wetzlar, Germany) inverted microscope equipped with a Leica DFC9000 GTC camera using LAS X software. Images were further processed (adjustment of brightness, scale bar) using ImageJ/Fiji [38].

### Statistical analysis

Data were graphed and statistically analyzed with the program Graphpad Prism (Graphpad Software, Boston, MA, USA) version 9.2.0.

## Results

### Establishment of immortalized rhesus macaque kidney cell lines

**Fig 1:**
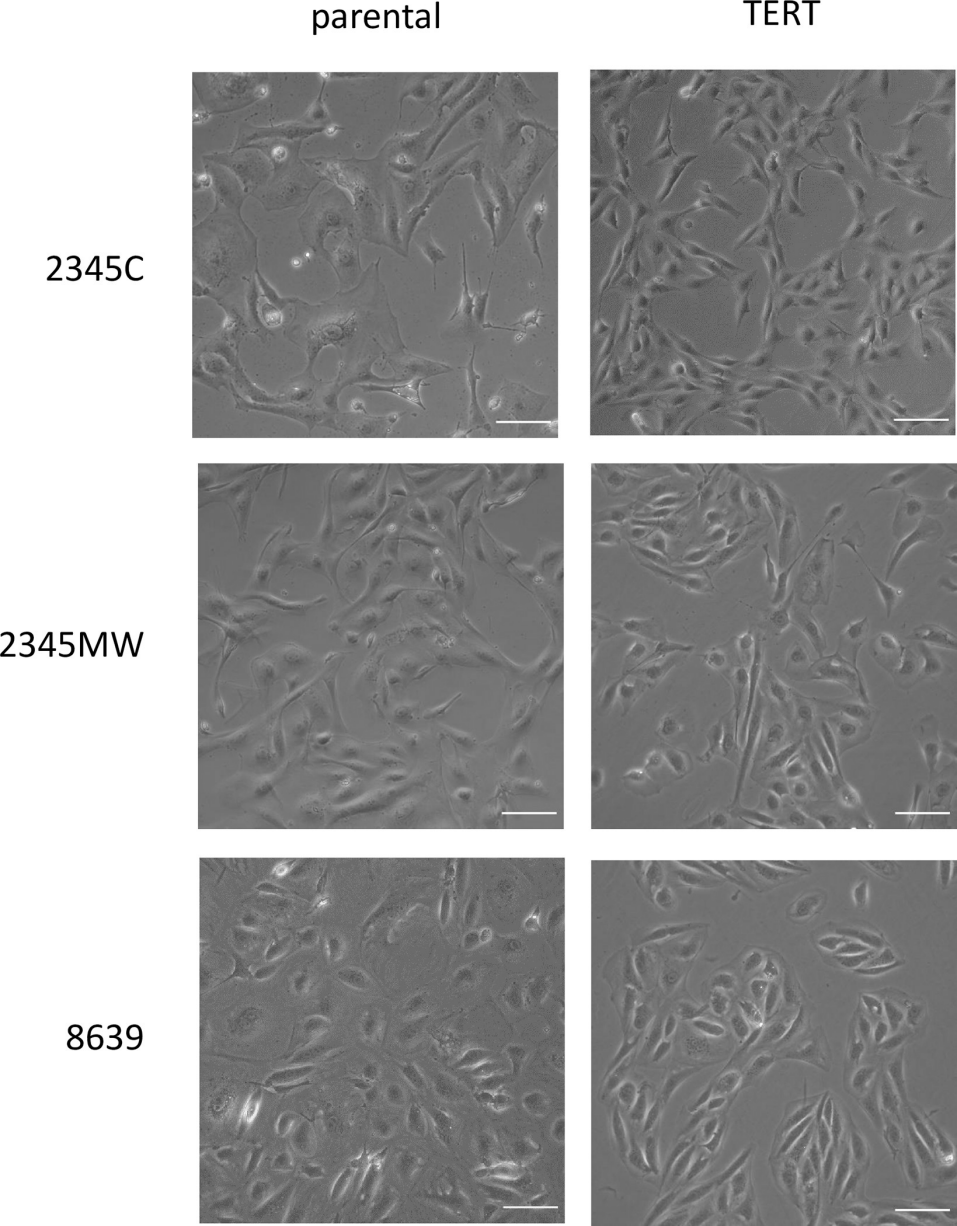
Morphology of parental and TERT immortalized rhesus kidney cells. Cells were seeded in 12-well plates and phase contrast images taken at 20x magnification. White scale bars indicate 100 μm.

**Fig 2:**
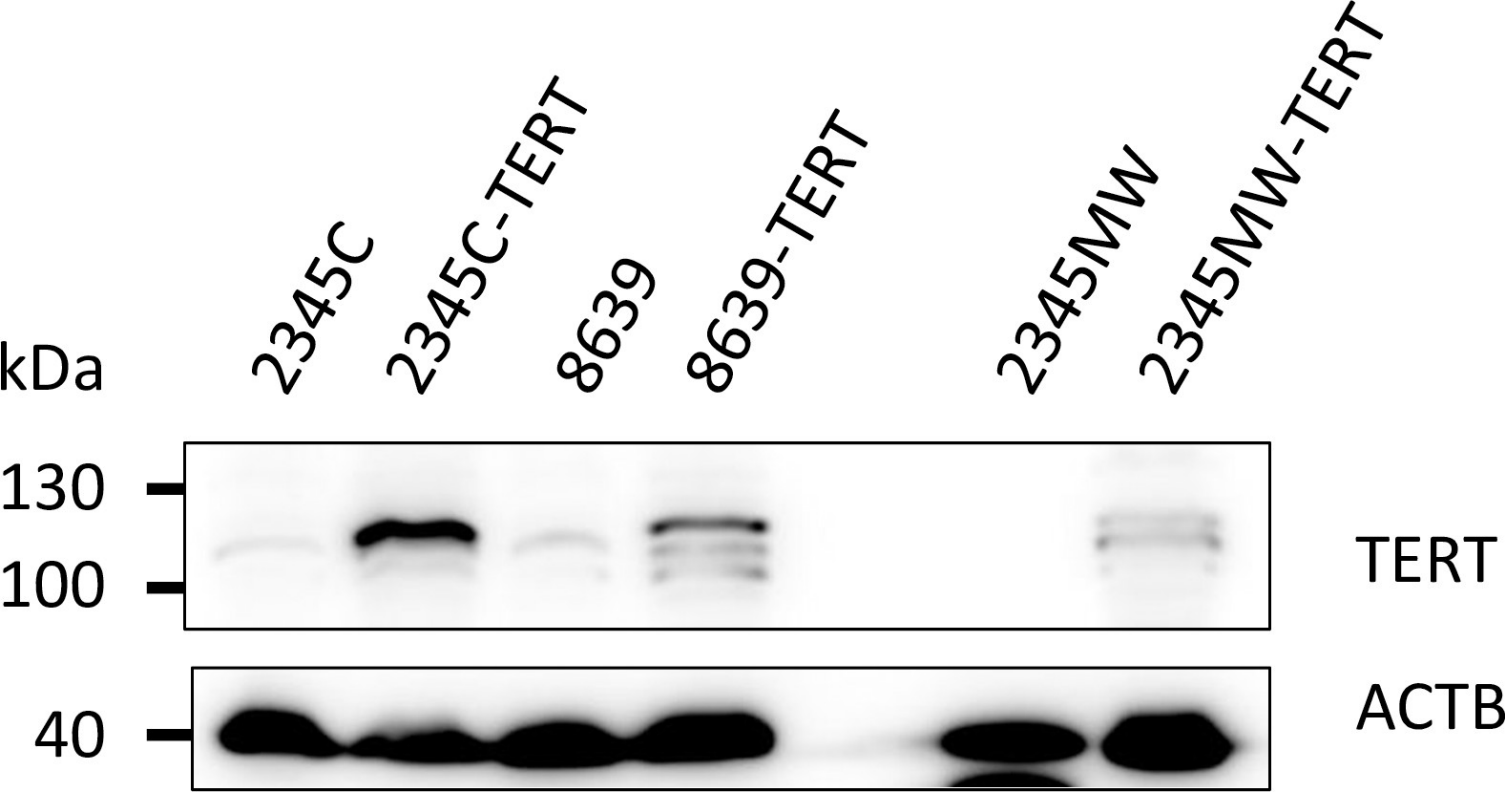
Immortalized kidney cells express the immortalization gene TERT. Lysates of parental and immortalized kidney cells were analyzed by western blot for expression of immortalization gene TERT. Detection of β-actin (ACTB) served as loading control. The results were confirmed in an independent experiment.

**Fig 3:**
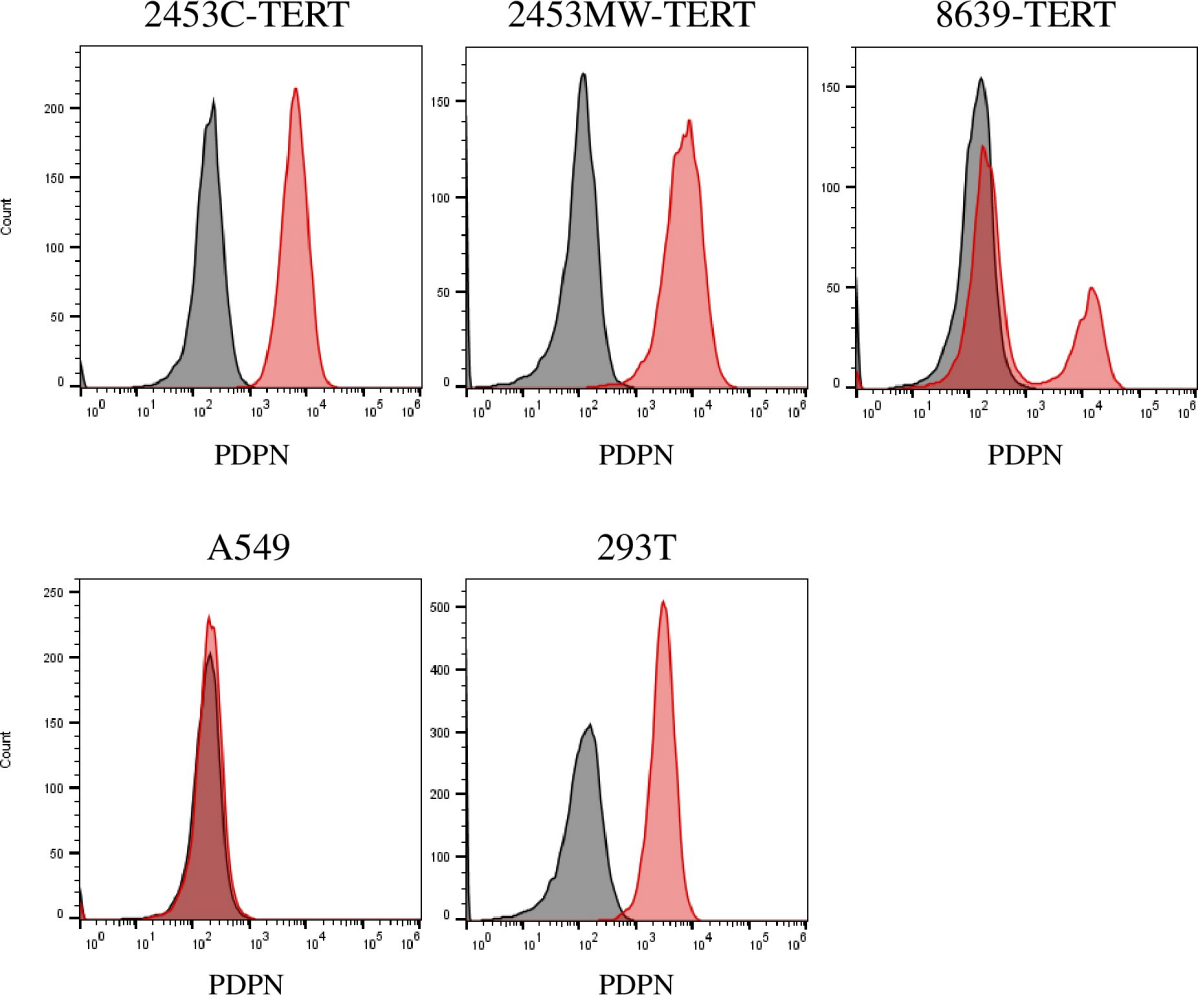
Immortalized kidney cells express the surface marker podoplanin. Cells were detached and stained with anti-podoplanin antibody (red) or isotype control antibody (black) and subjected to flow cytometry. A549 cells served as negative control while 293T cells were used as positive control. Cells shown in histograms were gated for singlets and intact cells. The results were confirmed in an independent experiment.

To establish cell lines from rhesus macaques, we dissociated kidney tissues from adult male (6 years, animal 2345) and female (9 years, animal 8639) animals and established separate individual primary cultures of kidney cells. The cell cultures were named *Macaca mulatta* kidney (MaMuK) cell lines MuMuK2345C, MaMuK2345MW, and MaMuK8639, respectively. Cell lines MuMuK2345C and MaMuK2345MW were established from distinct kidney tissue samples of the same male animal. After several passages cells were transduced with the human TERT gene, which has been shown to immortalize cells [9, 10]. For efficient transduction, an HIV-1-based lentiviral system was modified to include a chimeric Gag protein to avoid TRIM5α-mediated restriction [23, 24]. By selection with hygromycin, resistant cell cultures were established. Compared to the parental cultures the transduced cultures were less heterogenous, cells had a smaller cell body (Fig 1) and showed faster growth, as determined by calculation of doubling times (Table 2) or revealed in a CFSE-based cell proliferation assay (S1 Fig). All cell lines displayed a spindle-like morphology as seen in mesenchymal cells. Cells were successfully passaged at least 30 times, during which the cell population doubled at least 70 times. Expression of TERT in transduced but not parental cells was confirmed by Western blot (Fig 2) and was stable over multiple passages (S2 Fig). Finally, flow cytometric analysis of TERT-positive cells revealed expression of podoplanin (PDPN) (Fig 3), a podocyte maker [39] that is also expressed in 293T cells [40], a human embryonic kidney cell line frequently used for biomedical research. In addition, a weak expression of the podocyte marker protein Nephrin [39] was detected by Western blot in all three cell lines (S3 Fig). Thus, we had obtained three TERT and PDPN expressing permanent rhesus macaque kidney-derived cell lines that were subjected to further analysis.

**Table 2:** Doubling times (h) of cell lines.

	*parental*	*TERT*
MamuK2345C	n.a.	38.4 ± 5.7
Mamuk2345MW	168.8 ± 52.2	28.3 ± 12.4
Mamuk8639	63.8 ± 29.7	20.8 ± 3.1

n.a. not analyzed; means and standard deviations from 3–7 measurements are shown.

### Immortalized rhesus macaque kidney cell lines have a functional IFN system

**Fig 4:**
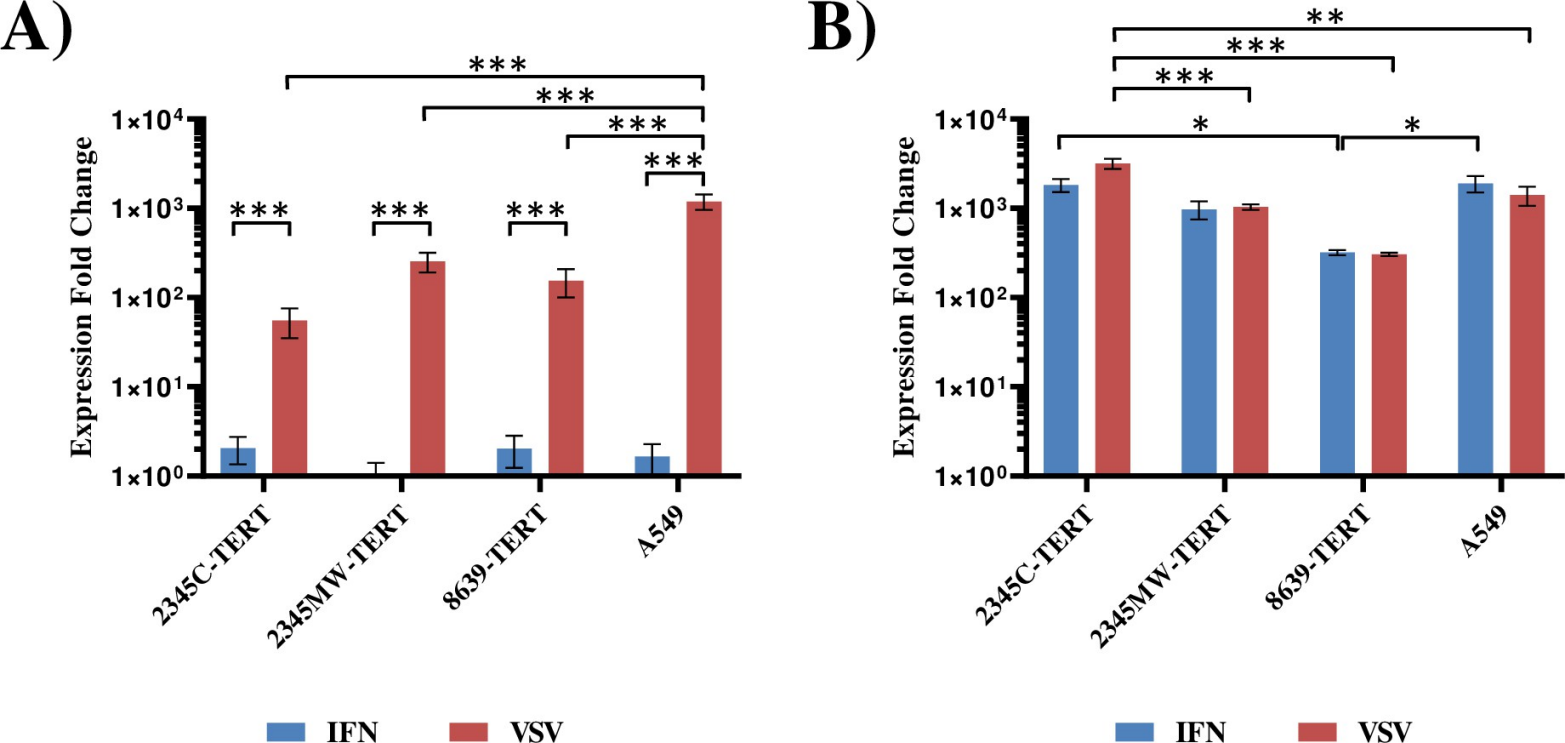
Immortalized kidney cells express IFNB1 and/or MX1 upon stimulation with IFN or VSV infection. Cells were seeded in 12 well plates and either treated with 100 U/mL pan-interferon or infected with VSV ncp* (MOI 0.1). Untreated cells served as control. After 24 h cells were harvested, RNA was isolated and analyzed by quantitative RT-PCR for expression of (A) interferon beta (IFNB1) or (B) MX1. Transcript levels were normalized against 18S rRNA transcript levels and expression fold change calculated with respect to control cells. The results of a single representative experiment performed in triplicates are shown and were confirmed in an independent experiment. Error bars indicate standard deviation. Results were assessed for statistically significant differences using ANOVA with Tukey’s correction: *, P ≤ 0.05; **, P ≤ 0.01; ***, P ≤ 0.001.

We next characterized whether the cells expressed interferon β (IFNB1) and the IFN-stimulated gene (ISG) MX1 in response to IFN or virus infection. For this, we treated the cells either for 24 h with 100 U/mL pan-IFN, a chimeric human IFN alpha, or vesicular stomatitis virus (VSV) ncp*, which strongly induces IFN [27]. Induction of IFNB1 or MX1 was analyzed by quantitative RT-PCR. The human lung cell line A549 was included as positive control, since this cell line has an intact IFN system [41]. Infection with VSV but not treatment with pan-IFN strongly induced IFNB1 expression in all rhesus macaque kidney cell lines analyzed and induction efficiency was roughly comparable to that measured for A549 cells (Fig 4A). Both, infection with virus and treatment with pan-IFN, strongly induced MX1 expression in the rhesus macaque kidney cell lines (Fig 4B). Levels of MX1 induction were lower than in A549 cells for all MaMuK cells but still more than 300-fold over background (Fig 4B). Collectively, these results indicate that all newly established cell lines had an intact IFN system.

### Immortalized rhesus macaque kidney cells lines are susceptible and permissive to virus infection

**Fig 5:**
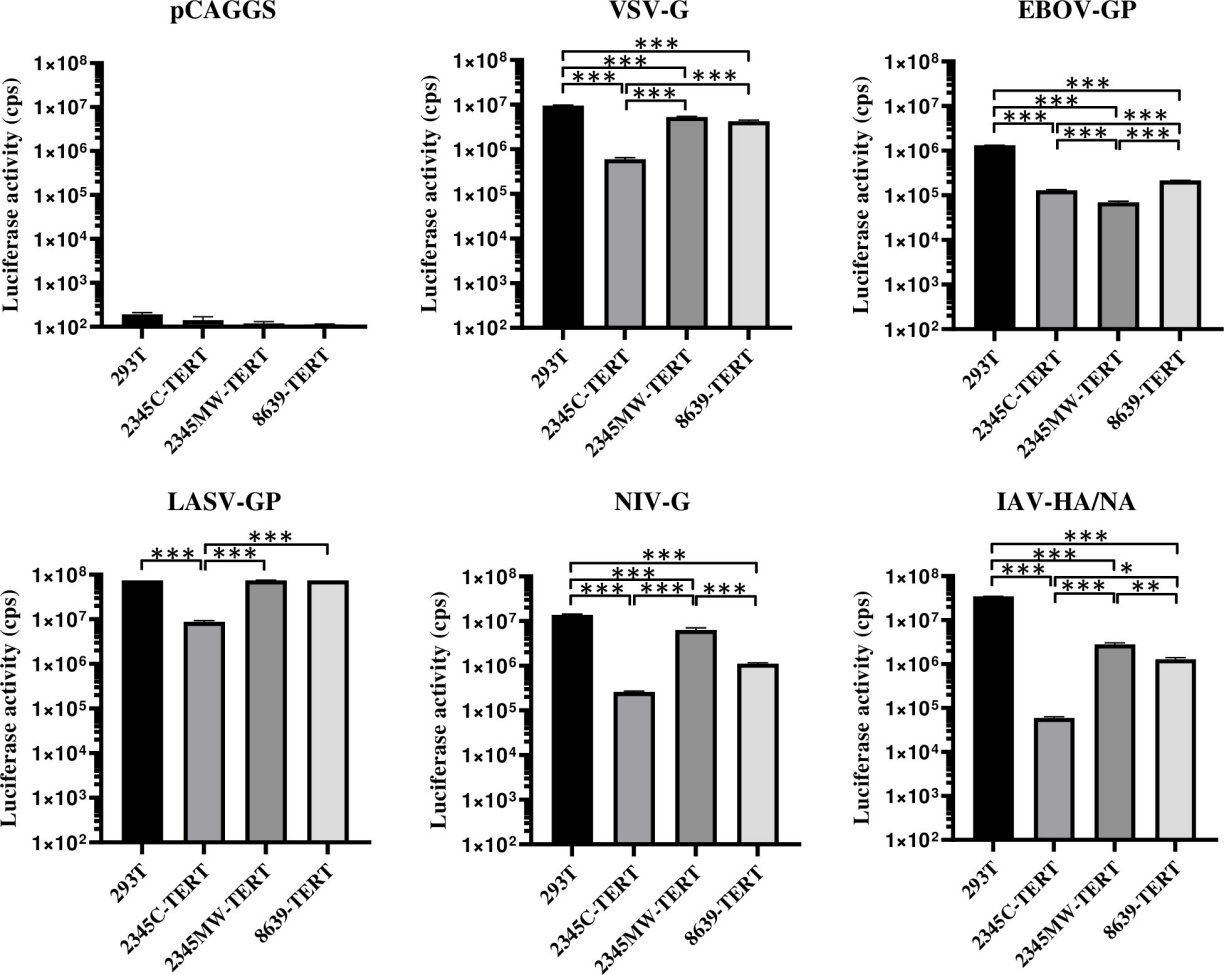
Immortalized rhesus kidney cells are susceptible to entry driven by diverse viral glycoproteins. Immortalized rhesus kidney cells were seeded in 96 well plates and transduced with MLV pseudoparticles encoding firefly luciferase and bearing the indicated viral glycoproteins. Transduction of 293T cells served as positive control while pseudoparticles without viral glycoprotein (pCAGGS) served as negative control. After 72 h cell lysates were harvested and luciferase activities determined. The results of a single representative experiment performed in triplicates are shown and were confirmed in an independent experiment. Error bars indicate standard deviation. Results were assessed for statistically significant differences using ANOVA with Tukey’s correction: *, P ≤ 0.05; **, P ≤ 0.01; ***, P ≤ 0.001.

We finally analyzed whether the cell lines were susceptible and permissive to viral infection. First, we chose a retroviral pseudotyping system to study susceptibility of cells to entry driven by the glycoproteins from Indiana vesiculovirus (VSV), Ebola virus (EBOV), Nipah virus (NIV), influenza A virus (IAV) or Lassa virus (LASV). Viral particles bearing no glycoprotein (pCAGGS) served as negative control while 293T target cells served as positive control since this cell line is known to allow entry driven by the above listed glycoproteins. The rhesus macaque kidney cell lines allowed for entry driven by all glycoproteins tested and entry efficiency was comparable to that measured for 293T cells (Fig 5).

**Fig 6:**
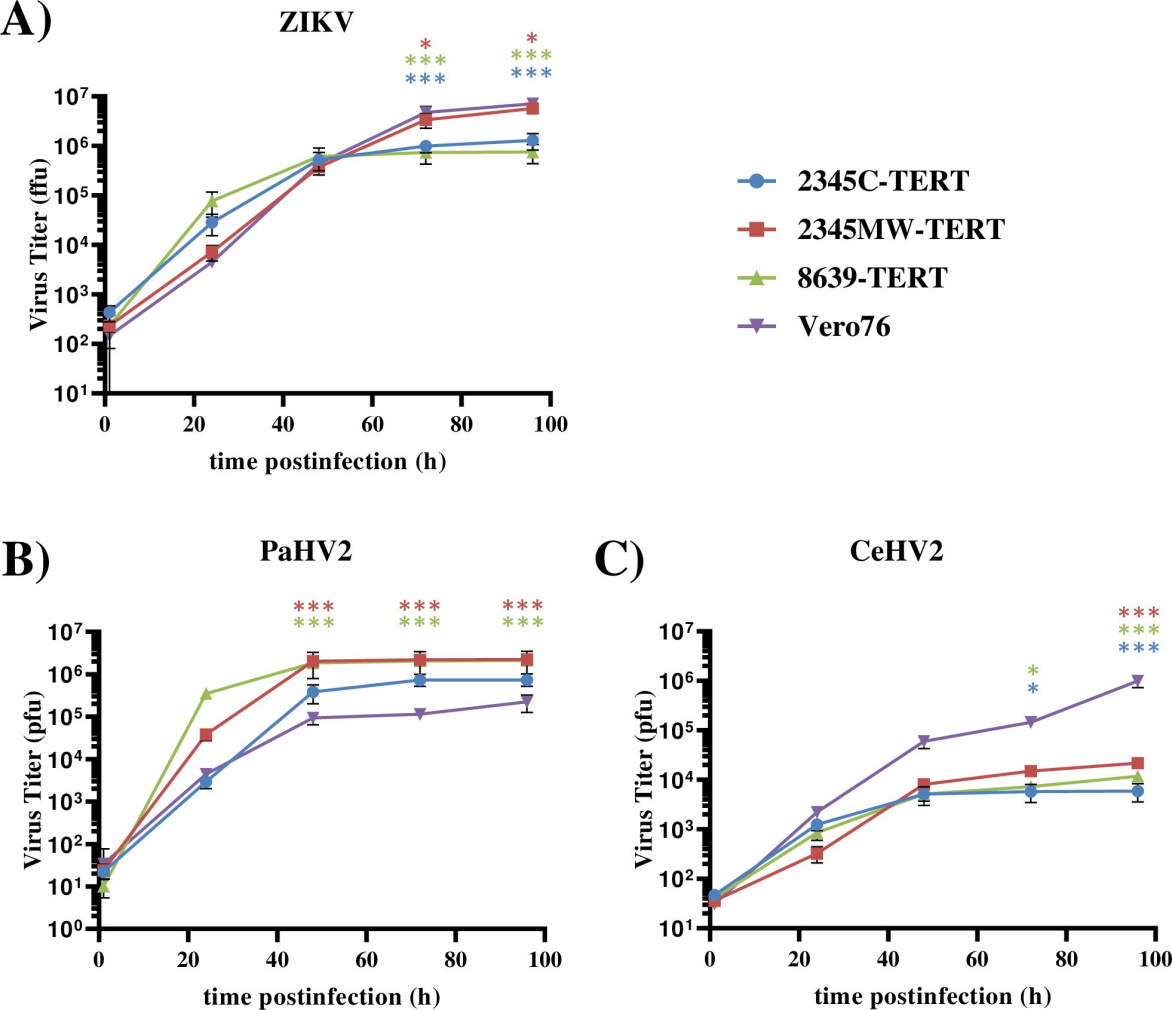
Immortalized rhesus kidney cells support growth of primate herpesviruses and Zika virus. Cells were seeded in 24 well plates and triplicate samples were infected with (A) Zika virus (ZIKV) strain SPH2015, (B) Papiine alphaherpesvirus 2 (PaHV2) or (C) Cercopithecine alphaherpesvirus 2 (CeHV2) at an MOI of 1. Vero76 cells served as positive control. Supernatants were harvested after 1, 24, 48, 72 and 96 h postinfection and infectious virus titer was determined by plaque assay (herpesviruses) or focus formation assay (ZIKV) on Vero76 cells. Virus titers are shown as plaque forming units (pfu) or focus forming units (ffu), respectively. The results of a single representative experiment are shown and were confirmed in an independent experiment. Error bars indicate standard deviation. Results were assessed for statistically significant differences with respect to Vero76 cells using ANOVA with Tukey’s correction: *, P ≤ 0.05; **, P ≤ 0.01; ***, P ≤ 0.001.

For analysis of permissiveness to viral infection, we employed selected RNA and DNA viruses. As RNA virus we used ZIKV, a human pathogen, and as DNA viruses we used the primate simplexviruses Papiine alphaherpesvirus 2 (PaHV2) and Cercopithecine alphaherpesvirus 2 (CeHV2), which naturally infect NHP. ZIKV replicated in all macaque cell lines with similar efficiency as compared to Vero cells (Fig 6A), which are routinely used to amplify ZIKV. The two herpesviruses showed a differential behavior. PaHV2 replicated to high levels in all macaque cell lines (Fig 6B), while CeHV2 replicated with markedly reduced efficiency in the rhesus macaque kidney cell lines as compared to Vero76 cells (Fig 6C).

## Discussion

We generated and characterized three cell lines derived from rhesus macaque kidney, which were immortalized by human TERT. These cells were susceptible to infection by diverse viruses, as determined with pseudotyped particles, and were permissive to infection with primate simplexviruses and ZIKV. Currently, a single rhesus macaque kidney cell line, LLC-MK2, is available from cell repositories. LLC-MK2 cells have epithelial morphology, whereas our cell lines display a spindle-like morphology as seen in mesenchymal cells. By flow cytometry, we could detect expression of the marker podoplanin (PDPN), placing them into the podocyte lineage according to single cell RNAseq data on kidney but could also reflect a dedifferentiated state [39, 42].

For analysis of viral infections, the status of the IFN system is of importance since sensing of viruses and subsequent induction of ISG expression can efficiently limit viral replication. As a consequence, cell lines that allow efficient amplification of diverse viruses, like Vero cells, frequently harbor a defective IFN system [43, 44]. While a more thorough study will be required to fully assess the IFN system in all its complexity, we could demonstrate that in the cell lines major pathways of the IFN system are intact, as evidenced by strong IFN beta induction upon VSV infection and strong induction of the ISG MX1 by IFN treatment or viral infection.

The rhesus macaque cells lines allowed entry driven by the glycoproteins from representatives of several virus families such as Rhabdoviridae, Filoviridae, Arenaviridae and Paramyxoviridae. Entry driven by the IAV (Orthomyxoviridae) hemagglutinin was also detected, but was less efficient than for human 293T cells. As a consequence, we expect that the cell lines should be susceptible to infection by diverse viruses. On a quantitative level, we note differences between individual cell lines in their susceptibility to infection, even for two cell lines (2345C, 2345MW) derived from the kidney of the same animal. These differences may result from heterogeneity of expression profiles even within a particular cell type in an organ as has been revealed in recent years by single cell transcriptomics [45, 46]. In addition, transfer of cells from tissue into cell culture will result in outgrowth of cells that grow best in the new cell culture environment [7]. Finally, retroviral transduction can affect genes close to the insertion site and result in additional selective pressure [47, 48].

Since the cell lines proved to be susceptible to several viruses, we also tested productive infection. Indeed, productive infection could be demonstrated for two primate herpesviruses, PaHV2 and CeHV2, as well as ZIKV (Flaviviridae). Virus titers were comparable or even higher than those attained in a highly permissive control cell line, Vero, despite a functional IFN system. Growth of CeHV2 in the rhesus macaque kidney cells was less efficient than in Vero cells, which may be due to species specific restrictions, as observed earlier and as also reported for Herpes simplex virus 1 and 2 [32, 49].

## Conclusions

We established three TERT-immortalized rhesus macaque kidney cell lines with an intact IFN system, which might support infection by diverse viruses, including primate herpesviruses and ZIKV. These cell lines could be valuable tools in comparative infection research or in translational research.

## Supporting information

S1 Fig CFSE-based cell proliferation assay. Cells were treated with CFSE and harvested directly after treatment (grey) or after 24, 48, 72 or 96 h (different shades of red). Untreated cells (white) served as control. Cells were analyzed by flow cytometry. Cells were gated for singlets and living cells, CFSE histograms were normalized to mode. Parental 2345C cells had ceased to grow at the time this assay was performed and were therefore not included in this assay.
(TIF)

S2 Fig TERT expression at different cell passages. Lysates of parental and immortalized kidney cells were analyzed by western blot for expression of the immortalization gene TERT. Detection of β-actin (ACTB) served as loading control. Passage number of individual parental or TERT-immortalized cell lines is indicated („p NUMBER“).
(TIF)

S3 Fig Nephrin expression in the macaque cell lines. Lysates of immortalized kidney cell lines were analyzed by western blot for expression of Nephrin (NPHS1) using a mouse monoclonal antibody (Santa Cruz, sc-377246). Kidney tissue from crab-eating macaques (Macaca fascicularis) served as positive control, A549 cells were used as negative control. Detection of β-actin (ACTB) served as loading control.
(TIF)

S4 Fig Original blot images. (PDF)

S1 Table Minimal data sets. (XLSX)
