# Supplementary figures and images for: Development of immortalized rhesus macaque kidney cells supporting infection with a panel of viruses
Source: PLoS One. 2023 May 5;18(5):e0284048. doi: 10.1371/journal.pone.0284048 (PMC10162512; doi:10.1371/journal.pone.0284048)

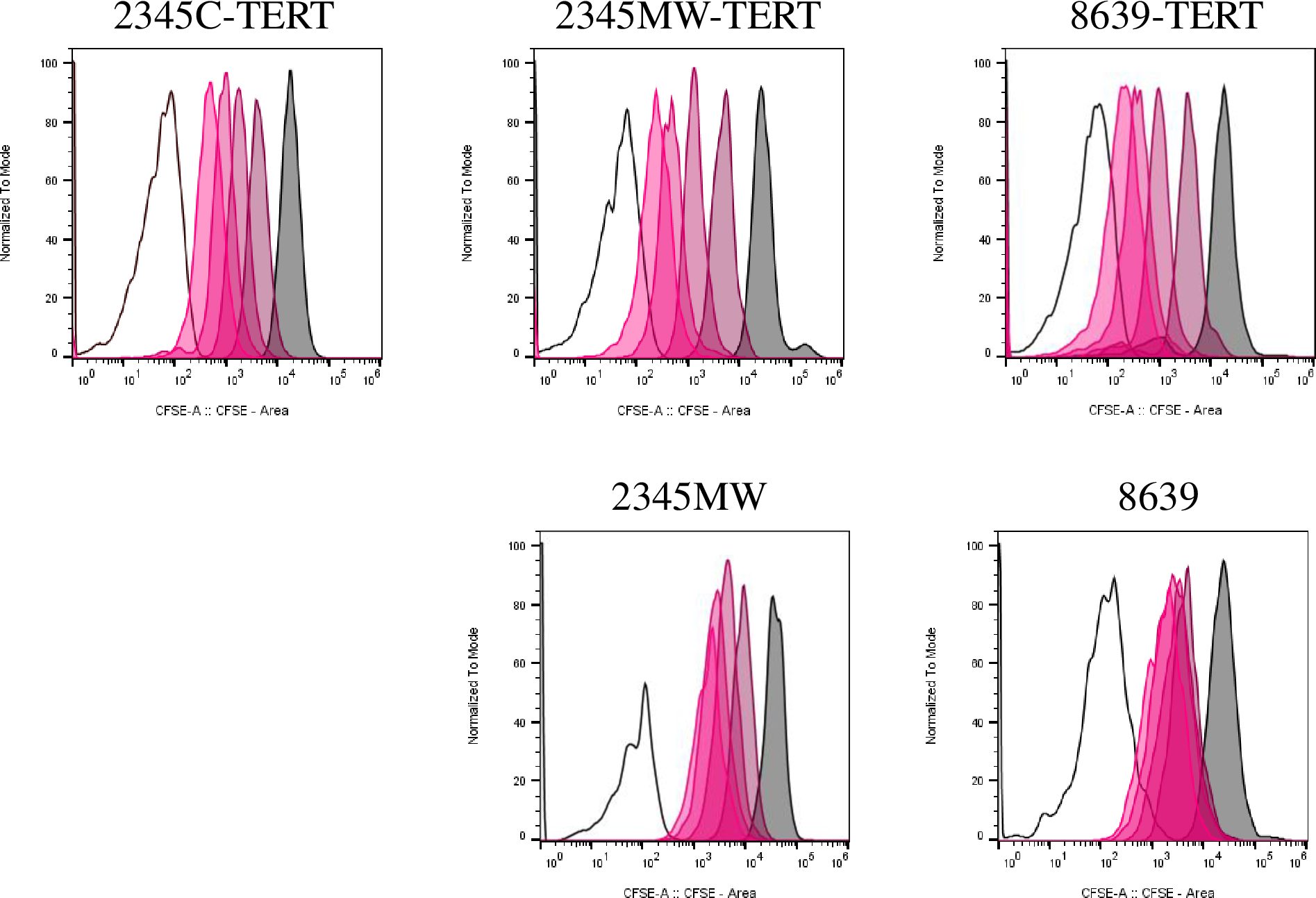

Supplement: S1 Fig — Cells were treated with CFSE and harvested directly after treatment (grey) or after 24, 48, 72 or 96 h (different shades of red). Untreated cells (white) served as control. Cells were analyzed by flow cytometry. Cells were gated for singlets and living cells, CFSE histograms were normalized to mode. Parental 2345C cells had ceased to grow at the time this assay was performed and were therefore not included in this assay. (TIF) [file pone.0284048.s001.tif]

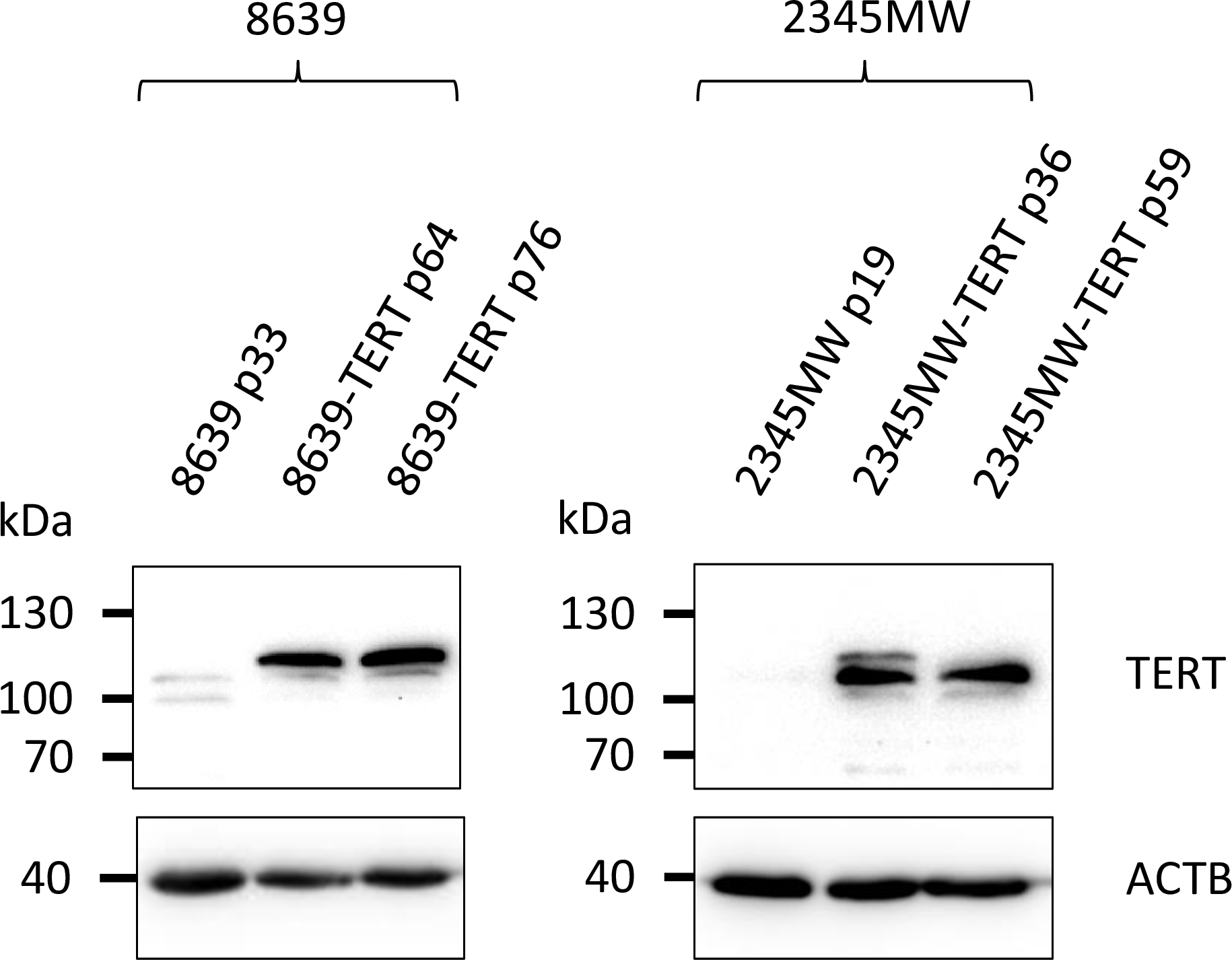

Supplement: S2 Fig — Lysates of parental and immortalized kidney cells were analyzed by western blot for expression of the immortalization gene TERT. Detection of β-actin (ACTB) served as loading control. Passage number of individual parental or TERT-immortalized cell lines is indicated („p NUMBER“). (TIF) [file pone.0284048.s002.tif]

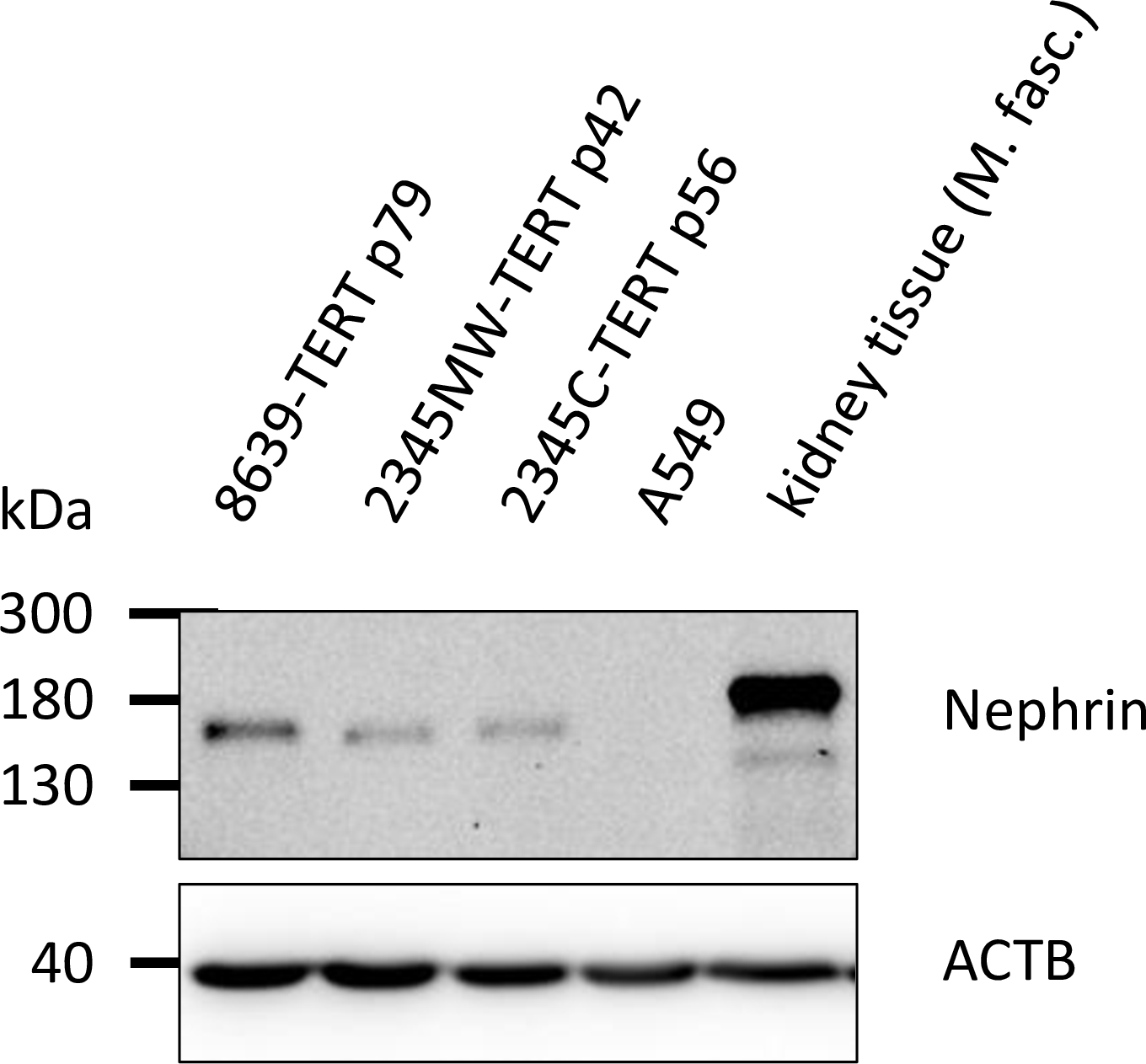

Supplement: S3 Fig — Lysates of immortalized kidney cell lines were analyzed by western blot for expression of Nephrin (NPHS1) using a mouse monoclonal antibody (Santa Cruz, sc-377246). Kidney tissue from crab-eating macaques (Macaca fascicularis) served as positive control, A549 cells were used as negative control. Detection of β-actin (ACTB) served as loading control. (TIF) [file pone.0284048.s003.tif]
