# Supplementary material for: Development of immortalized rhesus macaque kidney cells supporting infection with a panel of viruses
Source: PLoS One. 2023 May 5;18(5):e0284048. doi: 10.1371/journal.pone.0284048 (PMC10162512; doi:10.1371/journal.pone.0284048)

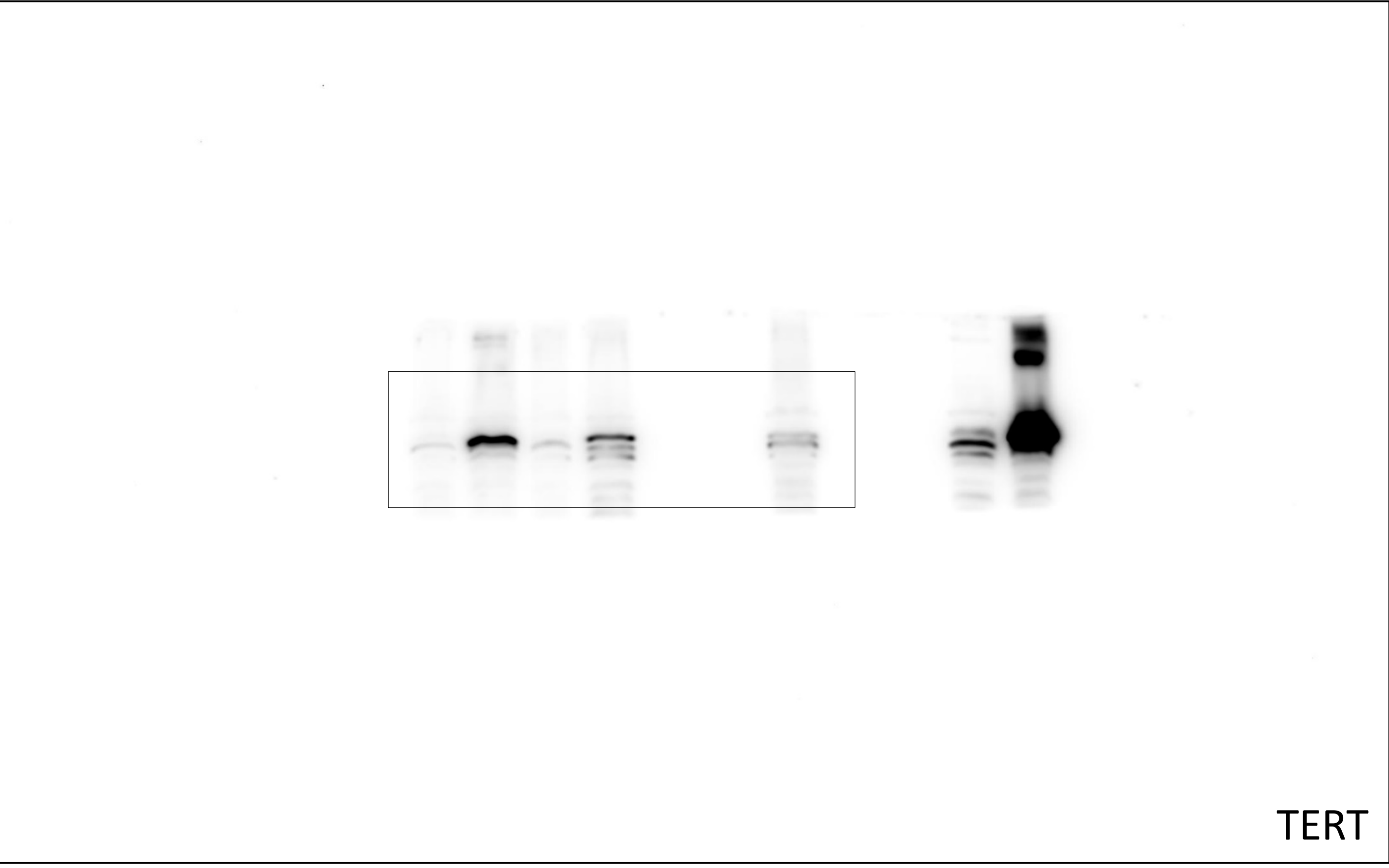

Fig. 2: Original immunostained images: Filter membrane was cut in two pieces and stained separately for TERT and β-actin

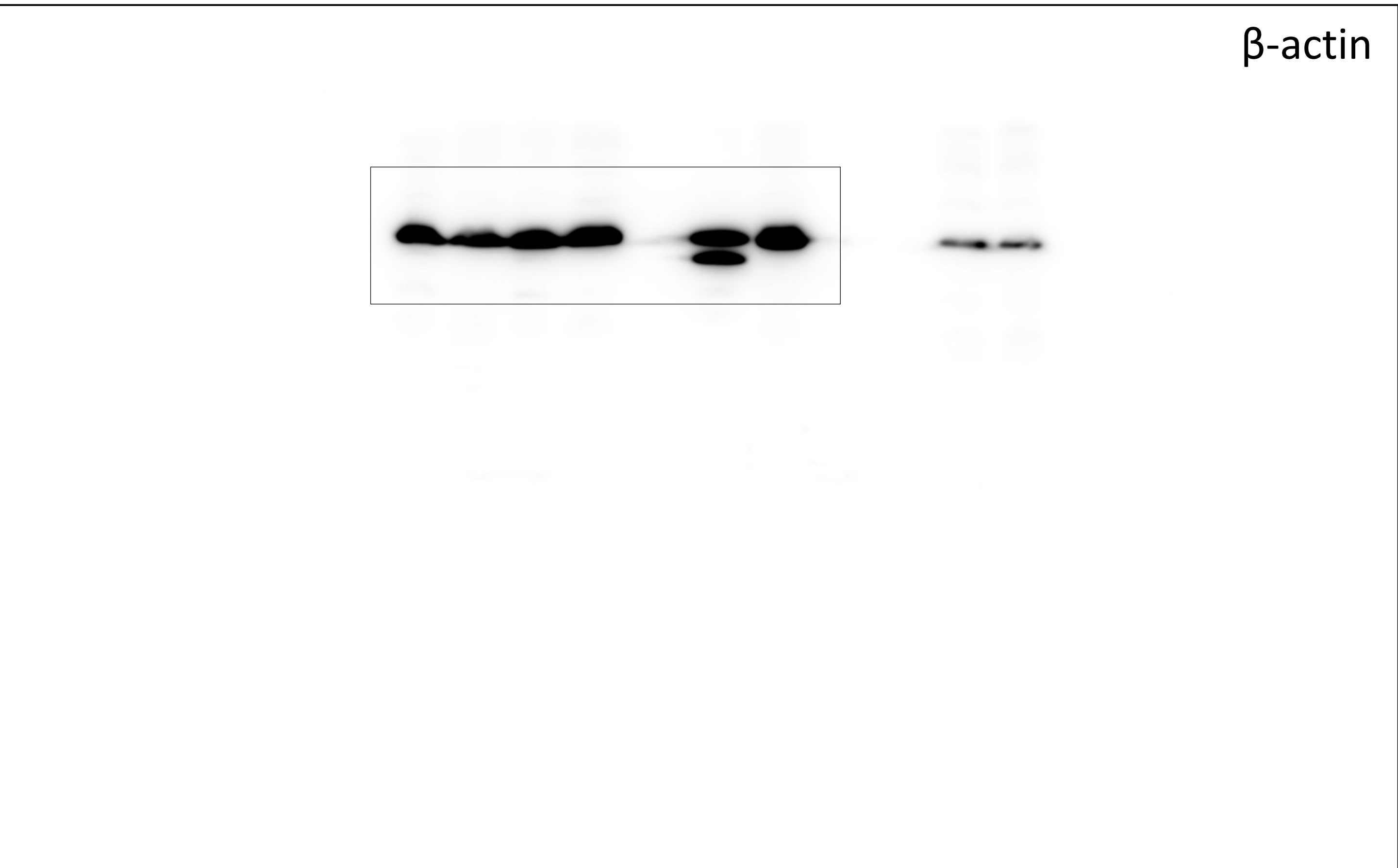

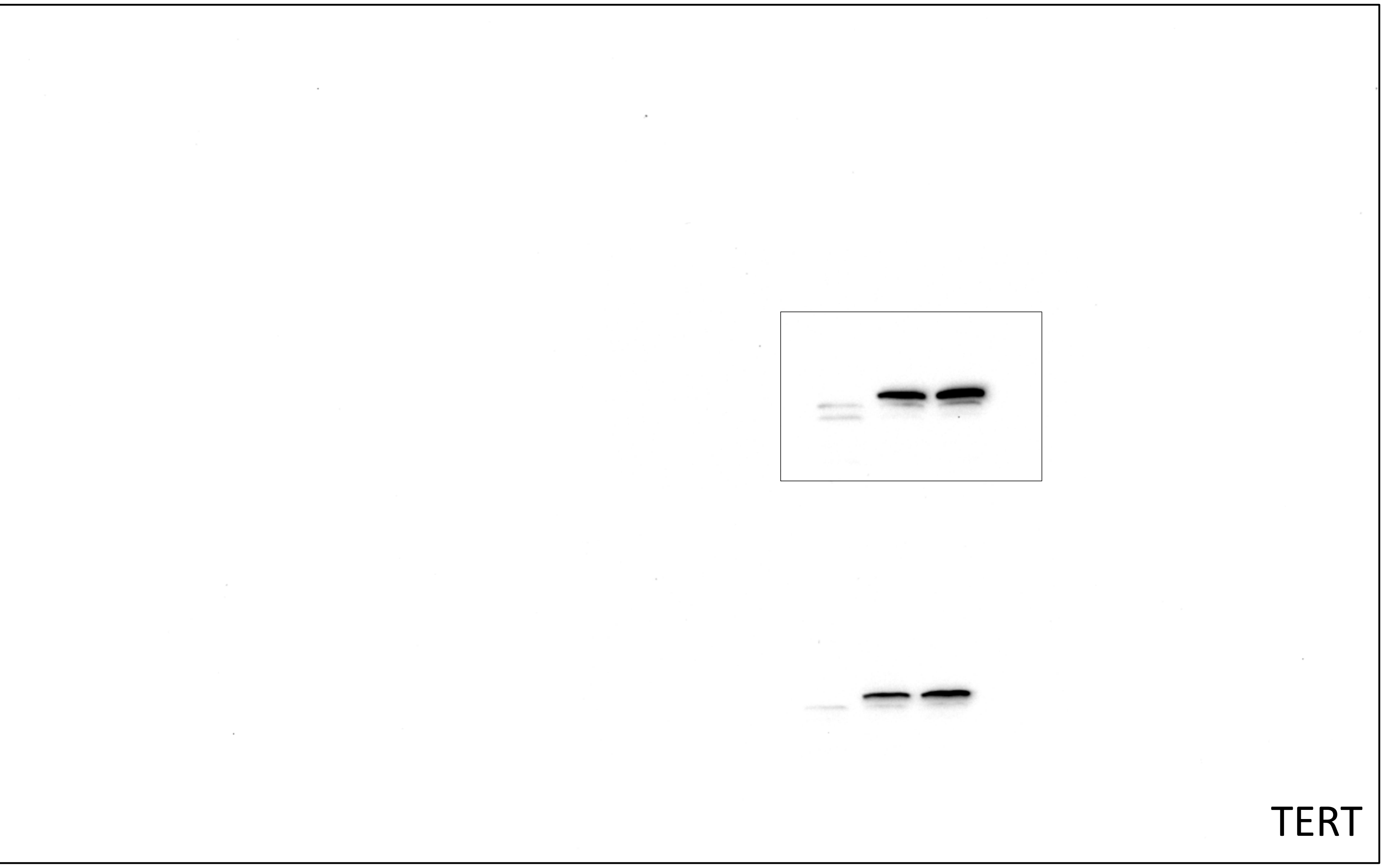

S2 Fig. 2: Original immunostained images of Mamuk8639 samples:
Filter membrane was cut in two pieces and stained separately

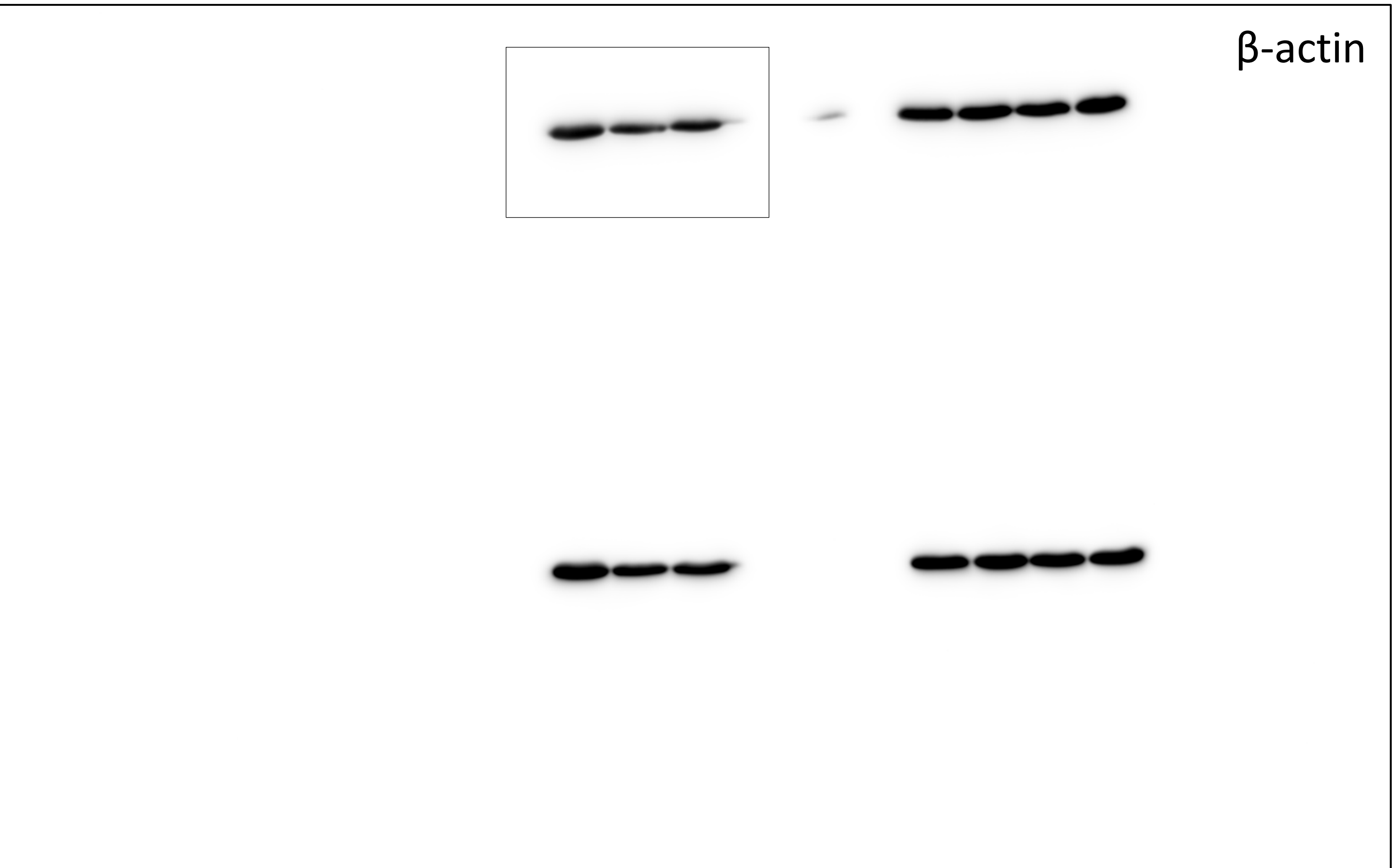

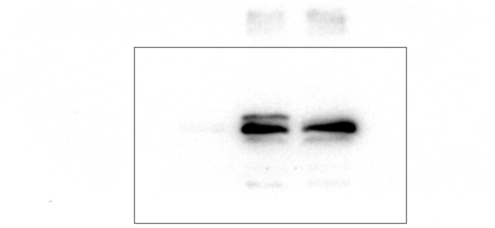

TERT

S2 Fig. 2: Original immunostained images of Mamuk2345MW samples: Filter membrane was cut in two pieces and stained separately

β-actin

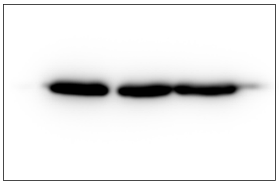

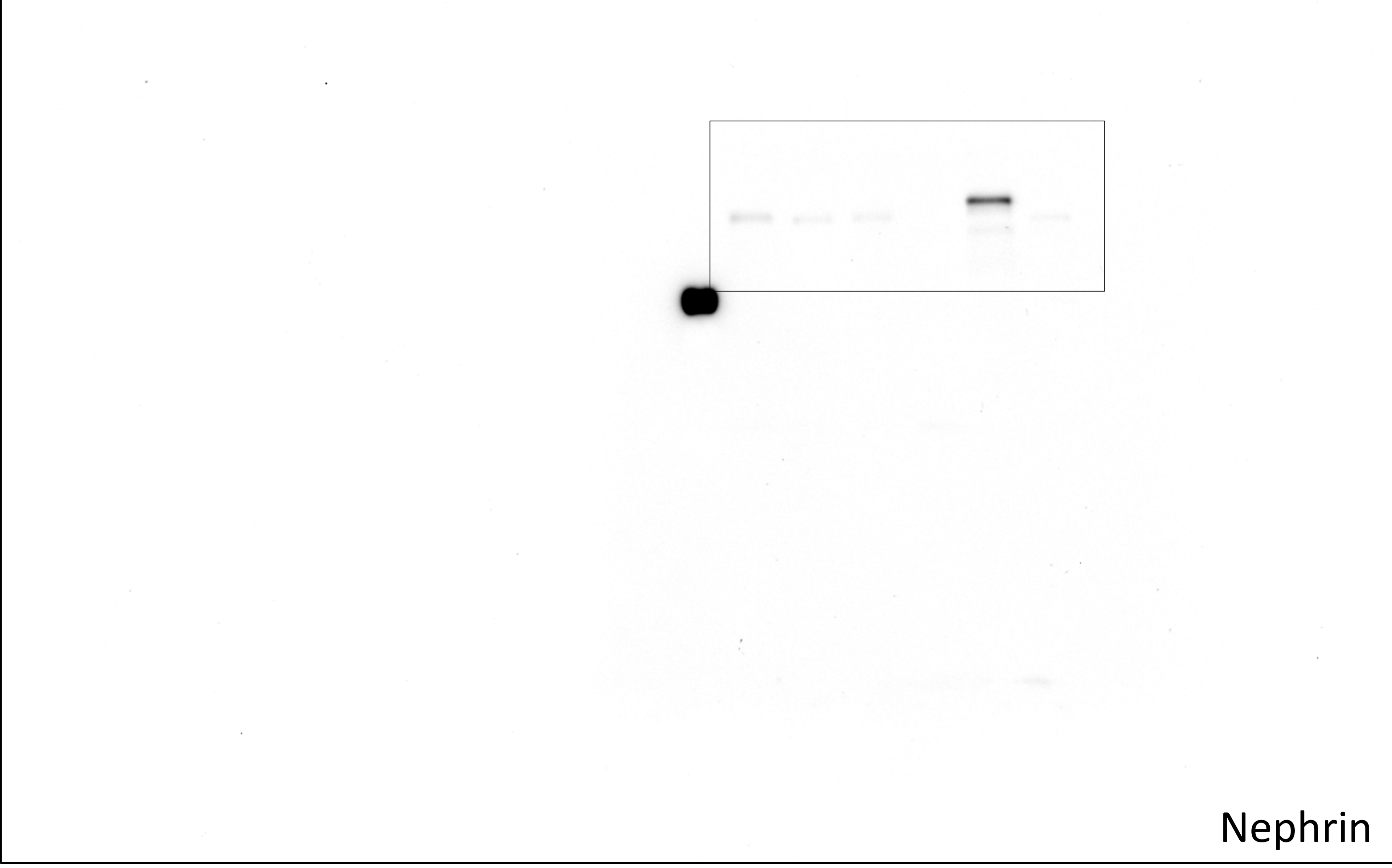

S3 Fig.: Original immunostained images: Separate filter membranes were stained for Nephrin and β-actin

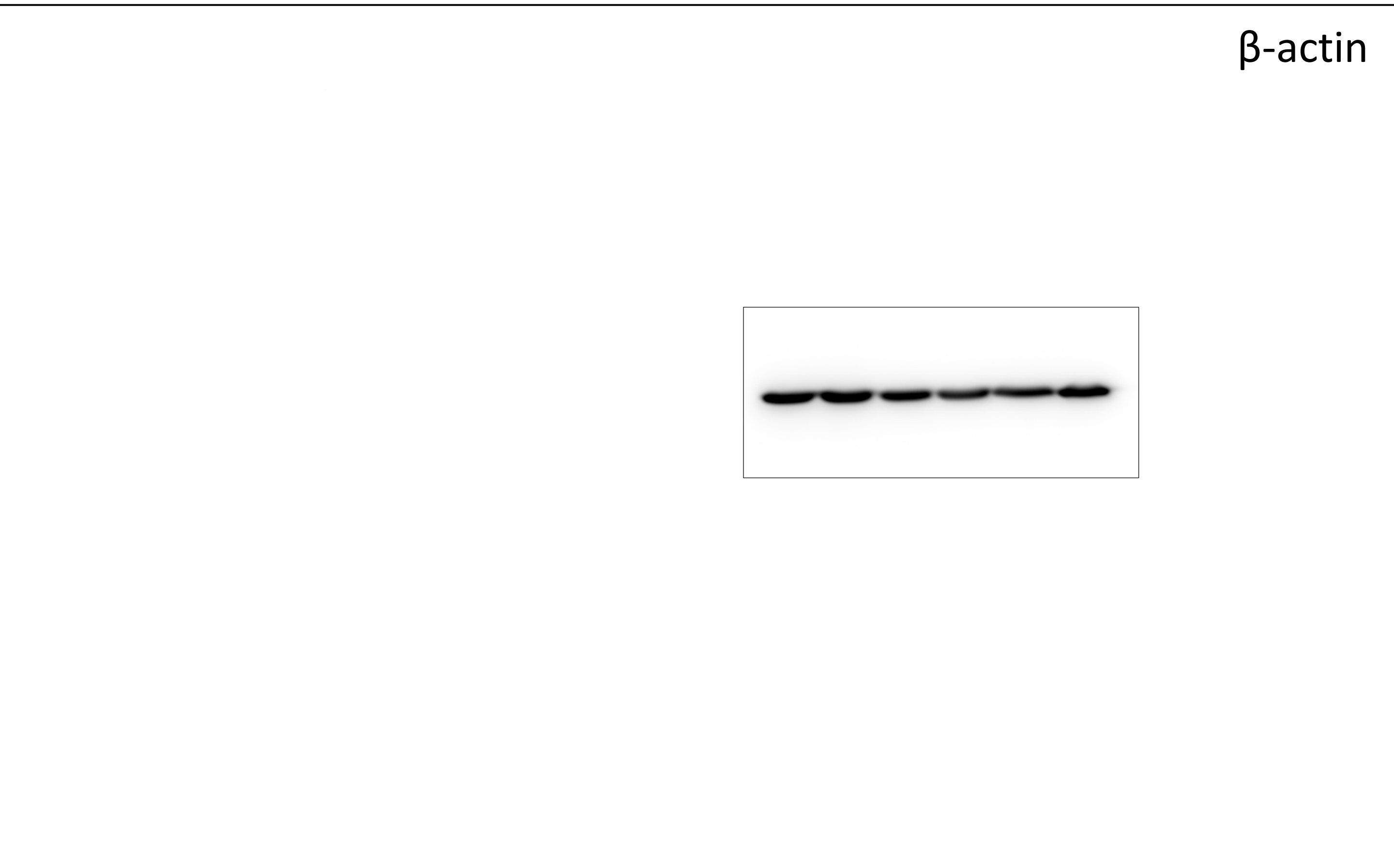

Supplement: S4 Fig — (PDF) [file pone.0284048.s004.pdf]
